# Zinc Modulation of Neuronal Calcium Sensor Proteins: Three Modes of Interaction with Different Structural Outcomes

**DOI:** 10.3390/biom12070956

**Published:** 2022-07-08

**Authors:** Viktoriia E. Baksheeva, Philipp O. Tsvetkov, Arthur O. Zalevsky, Vasiliy I. Vladimirov, Neonila V. Gorokhovets, Dmitry V. Zinchenko, Sergei E. Permyakov, François Devred, Evgeni Yu. Zernii

**Affiliations:** 1 Institut Neurophysiopathol, INP, Faculté des Sciences Médicales et Paramédicales, CNRS, Aix Marseille Université, 13005 Marseille, France; vbaksheeva@belozersky.msu.ru (V.E.B.); philipp.tsvetkov@univ-amu.fr (P.O.T.); francois.devred@univ-amu.fr (F.D.); 2 Belozersky Institute of Physico-Chemical Biology, Lomonosov Moscow State University, 119992 Moscow, Russia; 3 Plateforme Interactome Timone, PINT, Faculté des Sciences Médicales et Paramédicales, Aix-Marseille Université, 13009 Marseille, France; 4 Shemyakin-Ovchinnikov Institute of Bioorganic Chemistry, Russian Academy of Sciences, 117997 Moscow, Russia; aozalevsky@ibch.ru (A.O.Z.); vladimirov@bibch.ru (V.I.V.); 5 Institute of Molecular Medicine, Sechenov First Moscow State Medical University, 119991 Moscow, Russia; gorokhovets_n_v@staff.sechenov.ru; 6 Branch of the Institute of Bioorganic Chemistry, Russian Academy of Sciences, 142290 Pushchino, Russia; zdv@bibch.ru; 7 Institute for Biological Instrumentation, Pushchino Scientific Center for Biological Research of the Russian Academy of Sciences, 142290 Pushchino, Russia; permyakov.s@gmail.com

**Keywords:** neuronal calcium sensors, EF-hand, zinc, Zn^2+^-binding proteins, recoverin, visinin-like protein-1, VILIP1, neurocalcin-δ, NCLD, GCAP1, GCAP2, isothermal titration calorimetry, differential scanning fluorimetry, dynamic light scattering

## Abstract

Neuronal calcium sensors (NCSs) are the family of EF-hand proteins mediating Ca^2+^-dependent signaling pathways in healthy neurons and neurodegenerative diseases. It was hypothesized that the calcium sensor activity of NCSs can be complemented by sensing fluctuation of intracellular zinc, which could further diversify their function. Here, using a set of biophysical techniques, we analyzed the Zn^2+^-binding properties of five proteins belonging to three different subgroups of the NCS family, namely, VILIP1 and neurocalcin-δ/NCLD (subgroup B), recoverin (subgroup C), as well as GCAP1 and GCAP2 (subgroup D). We demonstrate that each of these proteins is capable of coordinating Zn^2+^ with a different affinity, stoichiometry, and structural outcome. In the absence of calcium, recoverin and VILIP1 bind two zinc ions with submicromolar affinity, and the binding induces pronounced conformational changes and regulates the dimeric state of these proteins without significant destabilization of their structure. In the presence of calcium, recoverin binds zinc with slightly decreased affinity and moderate conformational outcome, whereas VILIP1 becomes insensitive to Zn^2+^. NCALD binds Zn^2+^ with micromolar affinity, but the binding induces dramatic destabilization and aggregation of the protein. In contrast, both GCAPs demonstrate low-affinity binding of zinc independent of calcium, remaining relatively stable even at submillimolar Zn^2+^ concentrations. Based on these data, and the results of structural bioinformatics analysis, NCSs can be divided into three categories: (1) physiological Ca^2+^/Zn^2+^ sensor proteins capable of binding exchangeable (signaling) zinc (recoverin and VILIP1), (2) pathological Ca^2+^/Zn^2+^ sensors responding only to aberrantly high free zinc concentrations by denaturation and aggregation (NCALD), and (3) Zn^2+^-resistant, Ca^2+^ sensor proteins (GCAP1, GCAP2). We suggest that NCS proteins may therefore govern the interconnection between Ca^2+^-dependent and Zn^2+^-dependent signaling pathways in healthy neurons and zinc cytotoxicity-related neurodegenerative diseases, such as Alzheimer’s disease and glaucoma.

## 1. Introduction

Calcium signaling regulates multiple aspects of cellular function, such as gene expression, metabolic pathways, cell growth, and survival. In neurons, Ca^2+^ is fluxed mainly in synaptic terminals by voltage-gated channels and metabotropic receptors. It plays the role of a secondary messenger, controlling many nervous system-specific processes, such as the reception of various stimuli, neurite outgrowth, neurotransmission, and synaptic plasticity underlying learning and memory [1]. Besides regulating normal cellular function, specific Ca^2+^ signals trigger apoptotic pathways resulting in neuronal cell death and the development of neurodegenerative disorders, such as Alzheimer’s disease (AD), Huntington’s disease, Parkinson’s disease, amyotrophic lateral sclerosis (ALS), and others [2,3]. The physiological outcome of each particular calcium signal in neurons critically depends on its localization, as well as the range and duration of the calcium elevation. The diversity of these outcomes is provided by specific neuronal Ca^2+^ sensor (NCS) proteins, which respond to the binding or release of Ca^2+^ by forming active conformers, capable of regulating signaling targets and modifying their function. The signaling activity of NCSs thereby adapts the sensitivity of a neuron to the wide range of calcium concentrations, and provides the diversity of cellular responses to Ca^2+^ signals [1].

Mammals express five subgroups of NCS proteins, including NCS1 (subgroup A), five visinin-like proteins (VILIP1-3, neurocalcin-δ (NCLD) and hippocalcin;subgroup B), recoverin (subgroup C), three guanylyl cyclase-activating proteins (GCAP1-3; subgroup D), and four K+ channel-interacting proteins with several splice variants (KChIP1-4; subgroup E) [1]. The localization of recoverin and GCAPs is restricted to the retina, where they play strictly defined roles in Ca^2+^-dependent regulation of visual transduction in photoreceptor neurons. The function of GCAPs is limited to the modulation of photoreceptor guanylyl cyclases (GCs), whereas recoverin controls rhodopsin desensitization by rhodopsin kinase (G-protein-coupled receptor kinase-1, (GRK1), and performs several less-studied activities, such as deactivation of photoreceptor phosphodiesterase (PDE6), buffering of intracellular calcium, and regulation of unknown targets outside outer segments of photoreceptors [4,5]. The proteins of subgroups A, B, and E exhibit much broader distributions and activities in the regulation of neuronal function. Interestingly, in the retina, subgroup B proteins, VILIP1 and NCLD, demonstrate different localization as compared to subgroup C and D proteins, being absent from photoreceptor cells but expressed in bipolar, amacrine, and retinal ganglion cells [6]. NCS1 binds more than 20 targets, including phosphatidylinositol 4-kinases and their regulators, dopamine D2 receptor (D2R) and kinases GRK1/GRK2, voltage-gated Ca^2+^ channels (VGCCs), and other proteins, thereby modulating the growth and survival of neurons, reception, neurotransmission, and synaptic plasticity [7]. VILIP1 regulates cAMP signaling, nicotinic acetylcholine receptor (nAChR) and purinergic receptor (P2X3) activities, while hippocalcin affects MAPK signaling and related pathways. In addition, NCS proteins of subgroup B, including NCLD, regulate various GCs [6,8]. KChIPs bind and regulate voltage-gated potassium channels of the Kv4 family. KChIP3 (DREAM or calsenilin) also interacts with more than 15 targets and acts as a transcription factor [7,9]. Notably, the dysregulation of NCS proteins, associated with alterations in their expression or mutations, has been found in several mental, neurodevelopmental, neurodegenerative, and neuro-ophthalmological disorders. Abnormalities in NCS1 expression and functions are associated with schizophrenia, bipolar disorder, autism, and Parkinson’s disease [10,11]. VILIP1 is found in amyloid plaques of patients with AD, and its increase in cerebrospinal fluid is considered as biomarker of the disorder. In addition, the accumulation of disulfide forms of VILIP1 is regarded as a hallmark of amyotrophic lateral sclerosis (ALS) [12,13,14]. Furthermore, NCLD contributes to the development of spinal muscular atrophy, mutations in the hippocalcin gene have been detected in patients with autosomal recessive primary isolated dystonia, KChIPs has been linked to the pathogenesis of AD and Huntington’s disease, while mutations in GCAPs are associated with human retinal dystrophies and glaucoma [15,16,17,18,19,20,21].

Structurally, NCSs are small α-helical proteins (about 20 kDa) containing four EF-hand motifs assembled in N-terminal (EF1–EF2) and C-terminal (EF3–EF4) domains. In all cases, EF1 is unable to bind Ca^2+^ due to the substitution of two metal-coordinating residues and the presence of proline in the Ca^2+^-binding loop. In addition, recoverin and VILIP1 have an inactivated EF4 with internal salt bridge, whereas KChIPs has non-functional EF2 with substitutions in the Ca^2+^-coordinating sphere. All NCS proteins, except for KChIPs, contain a co-translational modification; i.e., the N-terminal myristoylation. The myristoyl group participates in membrane binding and accomplishes several structural functions. In Ca^2+^-free NCSs, it is buried inside the core due to interaction with a specific set of hydrophobic resides, and acts as an integral chaperone reshaping each of these homologous proteins into a unique structure. NCSs of subgroups B and C undergo a Ca^2+^-myristoyl switch, i.e., Ca^2+^-induced reversible exposure of the myristoyl group and the hydrophobic residues, intended for membrane binding and target recognition, respectively [22]. In Ca^2+^-bound NCS1, the myristoyl group is also extruded, but attaches the membrane irreversibly, and the subsequent release/binding of calcium regulate only the exposure of the target binding site [23,24]. In GCAPs, the myristoyl group does not undergo the switch, although in GCAP2 it can penetrate the membrane. Instead, in GCAP1, it is involved in Ca^2+^-dependent conformational transitions between GC-activating and GC-inhibiting states of the protein [25]. In most of the NCSs, the correct target recognition is supported by unique C-terminal segments arranging the shape of the binding site and/or providing additional contacts with the target [26,27,28,29,30]. All these structural features govern the Ca^2+^-affinity of each NCS protein, which, taken together, covers the entire range of Ca^2+^-signals in neurons (K_D_ values for calcium vary from 60–200 nM for GCAPs and 90 nM for NCS1 to 1.2 µM for VILIP1 and 17 µM for recoverin [26,27,31,32,33]). Besides the number of functional EF-hands and the nature of the residues in the Ca^2+^-binding loop, the affinity to calcium is determined by the energy cost of conformational changes associated with its coordination, and is affected by the presence of cellular membranes, individual phospholipids, and/or target proteins [22,34].

Previously, we have demonstrated that NCS proteins of subgroups A (NCS1) and C (recoverin) bind zinc [35,36,37]. This finding is important since zinc is highly abundant in neurons and exhibits both physiological activity and pathophysiological effects in neurodegenerative disorders, such as AD, ALS, as well as in neuro-ophthalmological diseases, including glaucoma and age-related macular degeneration [38,39,40,41,42]. In proteins, Zn^2+^ binding can play a structural, regulatory, or co-factor function, or destabilize proteins and promote their aggregation with formation of insoluble deposits [38,41,43,44,45]. Neurodegenerative processes are known to be associated with the aggregation of NCS proteins [12,14], which, in the case of recoverin and NCS1, can be promoted by zinc [35,36,37]. Recoverin and NCS1 exhibit different structural and functional effects in response to Zn^2+^ binding. Thus, in the case of recoverin, zinc decreases the melting temperature and increases membrane affinity of the Ca^2+^-saturated protein, while in NCS1, it enhances thermal stability without affecting its membrane association [35,36]. Considering these observations, we hypothesized that Zn^2+^ binding is a common feature of NCS family and serves as complementary factor for diversification cellular activities of these proteins. To test this hypothesis, we examined Zn^2+^-binding properties of five proteins belonging to different subgroups of the NCS family, namely, VILIP1 and NCLD (subgroup B), recoverin (subgroup C), as well as GCAP1 and GCAP2 (subgroup D), by a set of biophysical techniques. The common property of these proteins is that they are all expressed in the retina, which contains unusually high concentrations of zinc [46]. Our data indicate that NCS proteins indeed possess different sensitivity to zinc, and exhibit different structural outcomes in response to its binding, which may be of high importance for their normal and pathological signaling function in neurons.

## 2. Materials and Methods

### 2.1. Preparation of NCS Proteins

Plasmids for bacterial expression of recoverin (in pET11d) and NCLD (in pET22b) were constructed in our previous studies [47,48]. GCAP1 and GCAP2 expression vectors were kindly provided by our colleagues from the team of Prof. K.-W. Koch in the University of Oldenburg (Oldenburg, Germany) [49]. Molecular cloning of VILIP1 into pET22b plasmids was performed based on total mRNA from bovine brain, using a pair of specific primers: 5′-GGG CAT ATG GGG AAA CAG AAT AGC AAA CTG GC-3′ (direct, NdeI) and 5′-GGG AAG CTT CAT TTC TGA ATG TCG CAC TGC-3′ (reverse, HindIII). Expression and purification of the myristoylated proteins was performed according to previously published procedures [47]. Myristoylation rate was assessed with analytical HPLC using a reversed-phase column, as described in [47], and was more than 95%. All proteins were subjected to a standardized decalcification procedure to obtain the apo-forms [36]. Proteins were dialyzed against the same buffer containing 20 mM Tris (pH 7.5), 100 mM NaCl, and 0.5 mM tris (2-carboxyethyl)phosphine (TCEP), aliquoted, and stored at −20 °C. All buffer components, unless stated otherwise, were from Sigma (St. Louis, MO, USA), Honeywell Fluka (Charlotte, NC, USA), or BioMedEx (Glasgow, UK).

### 2.2. Isothermal Titration Calorimetry (ITC)

The binding of Zn^2+^ to NCS proteins was analyzed using iTC200 (MicroCal, Los Angeles, CA, USA) and PEAQ-ITC (Malvern Panalytical, Malverm, UK) isothermal titration calorimeters, as described previously [37]. All measurements were performed at 25 °C. Running buffer contained 20 mM Tris-HCl (pH 7.5), 100 mM NaCl, and 0.5 mM TCEP. The concentrations of proteins in the calorimetric cell were as follows: 70 µM apo-recoverin, 40 µM Ca^2+^-recoverin, 100 µM VILIP1, 50 µM GCAP1, and 25 µM NCLD. Concentration of ZnCl_2_ in the titrating syringe was 1.5 mM in all cases, with the exception of NCLD, where it was 0.75 mM. The proteins were titrated with 19 × 2 µL injections of ZnCl_2_ solution, with the exception of VILIP1, which was titrated with 38 × 1 µL injections. To control for dilution heat, ZnCl_2_ solution was titrated into the running buffer without the protein, and the resulting curve was subtracted from the experimental curve. The data were analyzed by MicroCal Origin 7.0 software (MicroCal, Los Angeles, CA, USA). The stoichiometry (N), constant (K_D_), enthalpy (ΔH), and entropy (ΔS) of the protein/ion interaction were calculated using the standard thermodynamic equations, based on the “one set of sites” model for recoverin, NCLD, and GCAP1, and the “two sets of sites model” for VILIP1.

### 2.3. Nano Differential Scanning Fluorimetry (nanoDSF)

Zn^2+^-induced conformational changes of NCS proteins were assessed using a label-free fluorimetric analysis, using a Prometheus NT.Plex instrument (NanoTemper Technologies, Munich, Germany) as previously described [36,50]. A total of 25 µM protein was loaded into NanoDSF grade capillaries (NanoTemper Technologies, Munich, Germany) in the presence of 25–200 μM Zn^2+^ with or without addition of 1 mM Ca^2+^. The samples were heated from 20 °C to 95 °C (recoverin, VILIP1, NCLD) or 110 °C (GCAPs) at a 1 °C/min heating rate. The excitation laser power was set at 10%. The ratio of innate tryptophan fluorescence intensities at 350 nm and 330 nm (I_350_/I_330_) was automatically determined by the pre-installed PR.ThermControl software (NanoTemper Technologies, Munich, Germany). Mid-transition melting temperatures (T_m_) for NCLD were calculated based on the temperature dependence of the first derivative of the I_350_/I_330_ fluorescence ratio, using the same software.

### 2.4. Thermal Shift Assay

Thermal shift fluorimetric assay of NCS proteins was performed according to the standard procedure described in [51]. First, 15 μM protein in buffer, containing 20 mM Tris-HCl (pH 7.5), 100 mM NaCl, 0.5 mM TCEP, was loaded with 250 μM CaCl_2_, 100 μM ZnCl_2_, both, or neither, and mixed with Sypro Orange dye 5000× DMSO stock (Sigma-Aldrich, St. Louis, MO, USA) to the final dye dilution rate of 5×. Subsequently, 20 μL samples were placed in 96-well MicroAmp reaction plates (Applied Biosystems, Waltham, MA, USA) and sealed with adhesive film. A 7500 Fast real-time PCR system (Applied Biosystems, Waltham, MA, USA) was used to monitor fluorescence during heat denaturation of the protein (20–99 °C, 1 °C/min heat rate). Thermal unfolding of the protein led to the binding of the dye to its exposed hydrophobic core, resulting in increased fluorescence intensity. Data analysis was performed using Mathematica software (Wolfram Inc., Champaign, IL, USA). Melting temperatures (T_m_) were determined based on the first derivative of temperature dependence of Sypro Orange fluorescence intensity.

### 2.5. Dynamic Light Scattering (DLS)

Measurements were performed using Zetasizer NanoZS (Malvern Instruments, Malvern, UK) at 25 °C or 42 °C. The dispersant viscosity was set to 0.8872 cP and the protein refractive index was set to 1.45. First, 25 μM NCS samples were analyzed in buffer containing 20 mM Tris-HCl (pH 7.5), 100 mM NaCl, and 0.5 mM TCEP, either with an addition of both 1 mM CaCl_2_ and 25–1000 μM ZnCl_2_, or neither. Samples were placed into 70 μL Ultra-Micro UV cuvettes (BrandTech, Essex, CT, USA) and subjected to 3 to 5 replicate measurements, each consisting of 10 runs of 10 s. The data were analyzed using Zetasizer software v.7.12 provided by the manufacturer (Malvern Instruments, Malvern, UK).

### 2.6. Structural Bioinformatics Predictions

Identification and ranking of putative Zn^2+^ binding areas was performed on the EF-hands of the NCS proteins, using the approach described previously [36]. Briefly, the significant parameters for Zn^2+^ coordination (distance and angle between the cation, coordinator, and one of the following atoms) were analyzed in PDB structures released no later than 7 December 2021. The possible range of coordinators was defined as a list of the following types of atoms: SG, ND1, NE2, OD1, OD2, OE1, OE2, OG, OG1, OH, and backbone oxygen (O). The maximal distance from the cation to the chelator was limited to 3 Å. The data were converted to the three-dimensional density with a scale from 0 to 100, yielding the reference density per coordinator type. EF-hand annotations were obtained from Uniprot and manually verified. Each EF-hand was scored independently. The reference density was projected to every coordinator in the EF-hand; areas that fell within VdW radii of neighboring atoms were subtracted. Volumetric data were visualized in PyMol (www.pymol.org; as of 1 June 2022). Analysis was performed on the following structures: X-ray: 6QI4, 4M2O, 5AER, 1OMV, 2R2I, 4M2Q, 4GUK, 4OV2, 5AFP, 4YI9, 4M2P, 5AEQ, 1G8I, 4MLW, 4YRU, 4YI8, 2HET, 1OMR, 1REC, 1BJF; NMR: 2I94, 2LCP, 1JBA, 1LA3, 1JSA. Plotting was performed using Matplotlib (University of Utah, Salt Lake, UT, USA) [52].

### 2.7. Data Analysis and Visualization

The data were gained from at least 3 independent measurements and analyzed with the mean standard error method. Data analysis and visualization was performed with SigmaPlot 12.5 software (Systat software, Chicago, IL, USA).

## 3. Results

### 3.1. Coordination of Zn^2+^ by NCS Proteins

**Figure 1:**
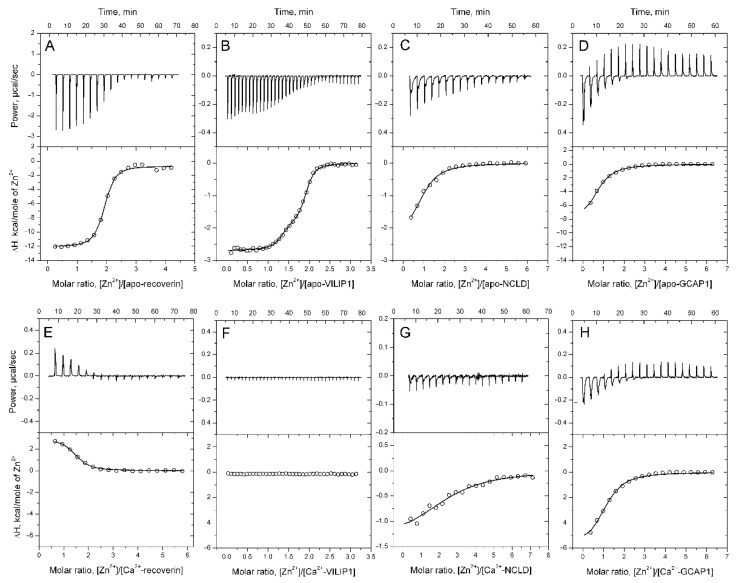
Binding of zinc ions to NCS proteins. Association between zinc ions and NCS proteins was monitored with ITC: (**A**) apo-recoverin; (**B**) apo-VILIP1; (**C**) apo-NCLD; (**D**) apo-GCAP1; (**E**) Ca^2+^-recoverin; (**F**) Ca^2+^-VILIP1; (**G**) Ca^2+^-NCLD; (**H**) Ca^2+^-GCAP1. Upper panels contain representative titration curves, and lower panels contain binding isotherms (open circles) and, where available, best fits (solid curves).

Previously, we identified recoverin as a Zn^2+^-binding protein [35]. In this study, we aimed to demonstrate that Zn^2+^ coordination is a common feature of the NCS family, and to further investigate the Zn^2+^-binding properties of NCS proteins, including recoverin. Thus, the analysis of the Zn^2+^-binding properties of recoverin, VILIP1, NCLD, GCAP1, and GCAP2 was performed using isothermal titration calorimetry (ITC). Proteins were titrated by ZnCl_2_ in the presence or in the absence of 1 mM CaCl_2_, and the obtained titration curves were fitted with the “one set of sites” model for recoverin, NCLD, and GCAP1, and the “two sets of sites” model for VILIP1 (Figure 1, Table 1). According to the ITC data, all analyzed NCSs, except GCAP2, bind Zn^2+^ (Figure 1, Table 1).

Apo-recoverin bound two zinc ions with K_D_ = 1 μM (Figure 1A), whereas Ca^2+^-recoverin coordinated one zinc ion with K_D_ = 2.7 μM (Figure 1E). In the first case, the binding was characterized by favorable enthalpy change (ΔH < 0), while in the second case, ΔH was positive, indicative of differences in the structure of the respective Zn^2+^-binding sites.

Similar to apo-recoverin, the apo-form of VILIP1 bound two zinc ions but with significantly different constants (43 nM and 1.7 μM) and stoichiometries (1.3 and 0.6) (Figure 1B, Table 1). The low stoichiometry of the second Zn^2+^-binding site suggests that it is located between the two subunits of a VILIP1 dimer, and thus, could potentially impact VILIP1 dimerization [53]. The presence of Ca^2+^ completely abolished Zn^2+^ binding to VILIP1, according to our ITC data (Figure 1F).

Apo-forms of NCLD and GCAP1 bound only one zinc ion with lower affinity (26 μM and 14 μM, correspondingly). While Ca^2+^ does not impact Zn^2+^ binding to GCAP1 (Figure 1D,H), it significantly alters the interaction between Zn^2+^ and NCLD, decreasing ΔH, and apparently changing its stoichiometry (Figure 1C,G).

Overall, we conclude that Zn^2+^ binding is a common feature of the NCS family, but each of these proteins demonstrates a unique mode of the interaction: recoverin and VILIP1 exhibits high-affinity Ca^2+^-dependent binding, NCLD demonstrates low-affinity Ca^2+^-dependent binding, whereas GCAP1 demonstrates low-affinity binding, which is unaffected by calcium.

### 3.2. Zn^2+^-Induced Conformational Changes in NCS Proteins

**Figure 2:**
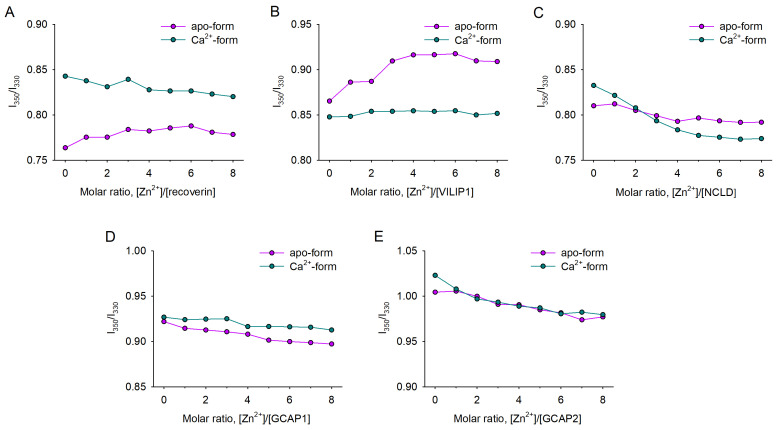
Zn^2+^-induced conformational changes of NCS proteins. Conformational shifts were detected by monitoring innate tryptophan fluorescence intensity at 350/330 nm (I_350_/I_330_) using the nanoDSF approach: (**A**) recoverin; (**B**) VILIP1; (**C**) NCLD; (**D**) GCAP1; (**E**) GCAP2. Fluorescence intensity ratios at 37 °C for apo- and Ca^2+^-bound NCS proteins were registered in the presence of increasing Zn^2+^ concentrations.

To assess whether Zn^2+^ binding is associated with structural transitions in NCS proteins, we next monitored the ratio of their tryptophan fluorescence intensity at 350 nm and 330 nm (I_350_/I_330_) in the presence of 1–8 fold molar excesses of ZnCl_2_ (25–200 μM) using differential scanning fluorimetry (nanoDSF). Incubation of apo-recoverin and apo-VILIP1 in the presence of increasing zinc concentrations induced an increase in the fluorescence intensity ratio (Figure 2A,B), suggesting that zinc coordination triggered a conformational shift in these proteins accompanied with exposure of tryptophan residues to the buffer. For both proteins, no further increase in the ratio was observed after a 3-fold molar excess of zinc, apparently due to the saturation of their high-affinity Zn^2+^-binding sites, which agrees with the ITC data. Meanwhile, other apo-NCSs (NCLD, GCAP1, and GCAP2) displayed a moderate decrease in I_350_/I_330_ in the presence of Zn^2+^, suggesting conformational changes, associated with burial of their tryptophan residues (Figure 2C–E).

In the presence of calcium, VILIP1 was completely unaffected by Zn^2+^, which is also in agreement with the ITC data (Figure 2B). In turn, Ca^2+^-saturated forms of recoverin, NCLD, and GCAPs displayed a gradual decrease in fluorescence intensity ratio, induced by Zn^2+^ (Figure 2A,D,E). Among these proteins, Ca^2+^-NCLD was the most susceptible to Zn^2+^, exhibiting a pronounced decrease in I_350_/I_330_, which was completed only upon saturation of its three high-affinity sites, as determined by ITC (Figure 2C).

### 3.3. Thermal Stability of Zn^2+^-bound NCS Proteins

**Figure 3:**
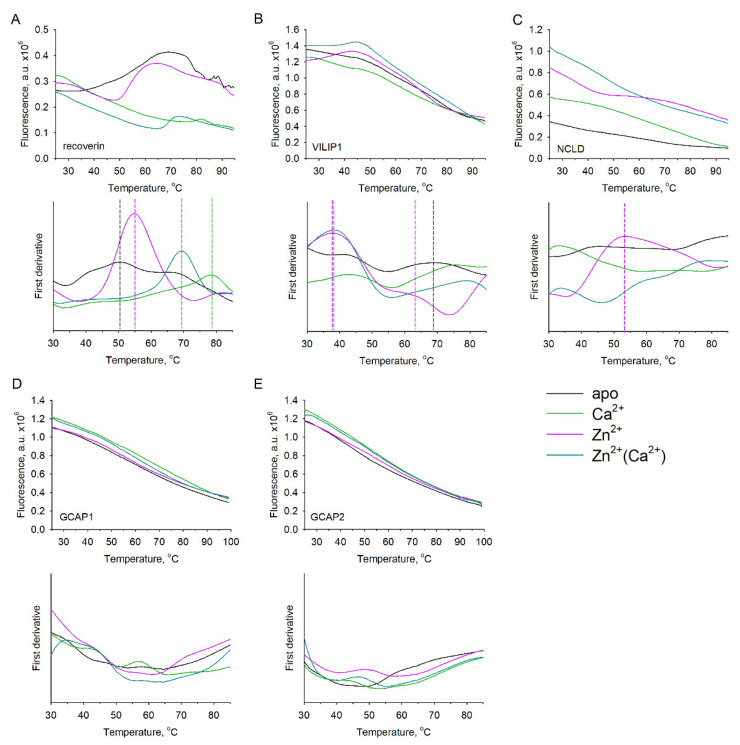
Thermal stability of NCS proteins in the presence of zinc. Thermal unfolding of metal-bound NCSs was monitored using the TS assay: (**A**) recoverin; (**B**) VILIP1; (**C**) NCLD; (**D**) GCAP1; (**E**) GCAP2. NCS samples were loaded with Sypro Orange hydrophobic fluorescent dye in the presence of Ca^2+^, Zn^2+^, both (“Zn^2+^(Ca^2+^)”), or neither (“apo”), and fluorescence intensity was monitored at 25–99 °C (top panels). Mid-transition melting temperatures (T_m_) were calculated based on the first derivative of each melting curve (bottom panels). Dashed lines represent heat denaturation points.

To analyze how Zn^2+^-induced conformational changes impact the overall stability of the NCS proteins, we monitored the thermal denaturation of their conformers using a thermal shift (TS) assay, or, alternatively, nanoDSF (by monitoring the temperature dependence of I_350_/I_330_). Zn^2+^ binding produced a moderate stabilizing effect on apo-recoverin, as its melting temperature (T_m_) increased from 50 °C to 55 °C. In contrast, the Ca^2+^-loaded form of the protein underwent slight destabilization in the presence of zinc (from 78 °C to 69 °C) (Figure 3A). Similarly, Zn^2+^ had only a mild effect on the thermal unfolding of apo-VILIP1 (T_m_ decreased from 69 °C to 64 °C, Figure 3B). For Ca^2+^-loaded conformers of VILIP1, T_m_ could not be determined, as it likely exceeded 90 °C, regardless of the presence of zinc. The small Zn^2+^-induced alterations, observed in the case of recoverin and VILIP1, likely reflect the conformational changes associated with the binding of this metal ion, rather than a non-specific destabilizing/pro-aggregative effect of Zn^2+^. Interestingly, the addition of Zn^2+^ resulted in the appearance of the second transition point at 38 °C in both conformers of VILIP1 (Figure 3B). A possible explanation for this transition is Zn^2+^-induced dissociation of VILIP1 dimers. This is consistent with the results of ITC studies, which suggested that the Zn^2+^-binding site is located at the intermolecular interface of the dimer (see above).

**Figure 4:**
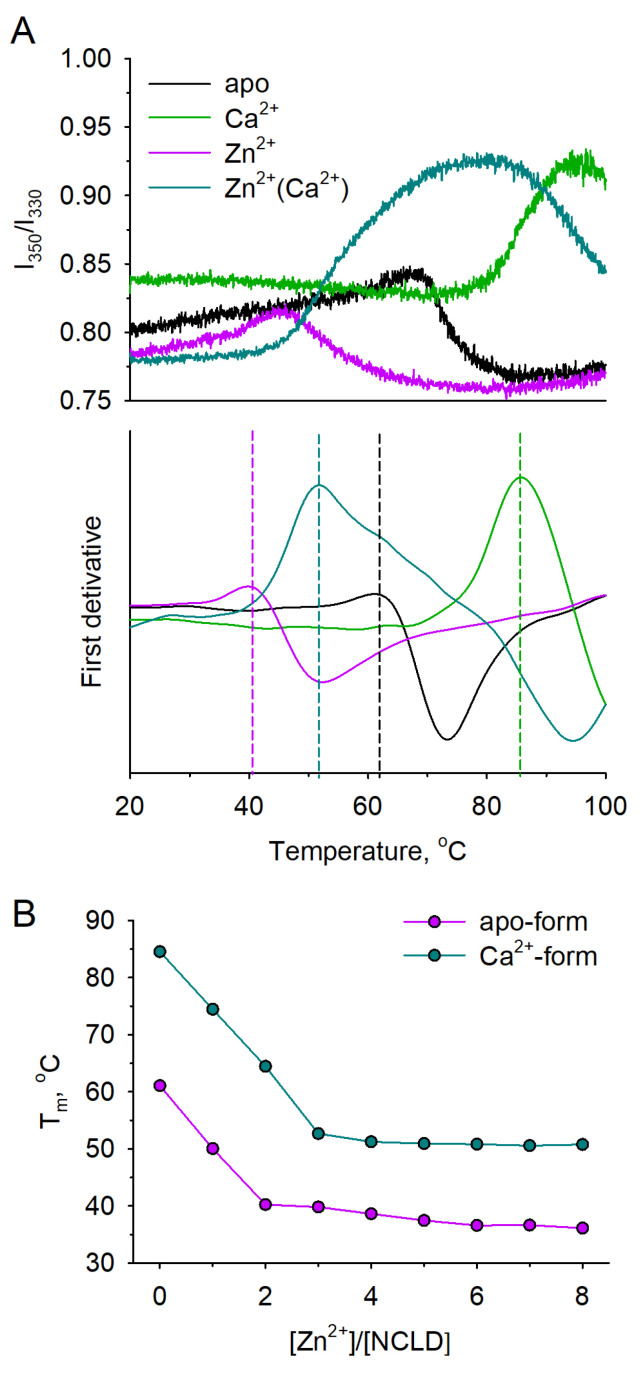
Thermal stability of NCLD in the presence of zinc. Thermal unfolding of NCLD was monitored using the nanoDSF approach by measuring temperature dependance of innate tryptophan fluorescence intensity ratio (I_350_/I_330_). (**A**) Top panel: melting curves of NCLD in the presence of saturating concentrations of Ca^2+^, Zn^2+^, both (“Zn^2+^(Ca^2+^)”), or none (“apo”); bottom panel: first derivative of the melting curve. (**B**) T_m_ of apo- and Ca^2+^-saturated NCLD in the presence of increasing Zn^2+^ concentrations. Dashed lines represent heat denaturation points.

In the case of NCLD and GCAPs, change in fluorescence was not detected in the observed temperature range by TS (Figure 3C–E); thus, the alternative method (nanoDSF) was applied. Both GCAPs were confirmed to not exhibit heat denaturation (data not shown) regardless of the presence of calcium or zinc, which agrees with the overall extreme structural stability of these proteins widely observed in previous studies [19,54]. Meanwhile, monitoring of heat denaturation of NCLD by nanoDSF revealed dramatic Zn^2+^-induced destabilization of both apo- and Ca^2+^-loaded forms of the protein, indicated by a decrease in apparent T_m_ from 61 °C to 39 °C, and from 84 °C to 52 °C, respectively (Figure 4B). It should be pointed out that thermal unfolding of NCLD conformers, marked by an increase in I_350_/I_330_, was followed by a decline in the tryptophan fluorescence ratio, suggesting immediate aggregation of the proteins upon denaturation (Figure 4A).

Based on these data, we conclude that Zn^2+^ binding has strikingly different impacts on the structure of NCS proteins, being almost indistinguishable in the case of GCAPs, regulating conformational states and/or the quaternary structure of recoverin and VILIP1, and producing a dramatic destabilizing effect on NCLD.

### 3.4. Zn^2+^-Dependent Aggregation and Oligomerization of NCS Proteins

**Figure 5:**
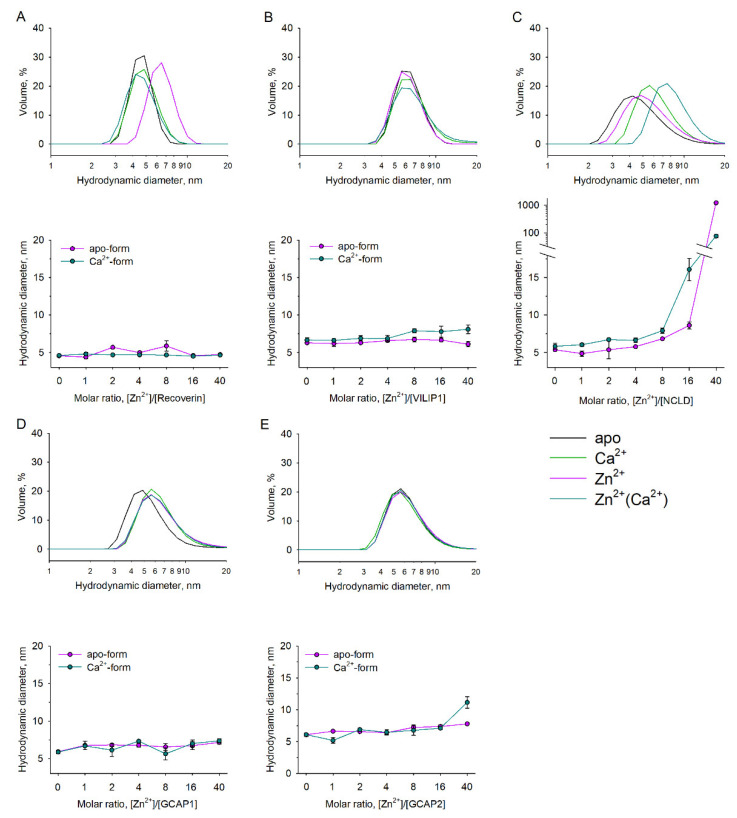
Zn^2+^-dependent oligomerization/aggregation of NCS proteins. Hydrodynamic diameter of NCS proteins was assessed by DLS: (**A**) recoverin; (**B**) VILIP1; (**C**) NCLD; (**D**) GCAP1; (**E**) GCAP2. All measurements were performed at 25 °C. Upper panels: representative size distribution curves for NCS in the presence of saturating concentrations of Ca^2+^, Zn^2+^, both (“Zn^2+^(Ca^2+^)”), or neither (“apo”). Bottom panels: mean hydrodynamic diameter values for apo- and Ca^2+^-bound NCS proteins in the presence of increasing Zn^2+^ concentrations. Standard error bars were plotted as a result of three independent measurements.

Our data strongly suggest that Zn^2+^ could affect the dimerization and aggregation of NCS proteins. To confirm this, we assessed the impact of zinc on their oligomeric state using the dynamic light scattering (DLS) method at different temperatures. In the case of recoverin, the binding of two zinc ions increased the hydrodynamic diameter of the proteins, regardless of the presence of calcium, from 4.6 nm to 5.7 nm, potentially reflecting Zn^2+^-induced dimerization (Figure 5A). This was not observed for Ca^2+^-loaded recoverin. VILIP1, GCAP1, and GCAP2 had initially larger hydrodynamic diameters (6.3, 5.9, and 6.1 nm for apo-forms, correspondingly), indicating that they existed as non-covalent dimers under our experimental conditions, which agrees with previously published data [53]. The dimeric states of these proteins were generally not affected by increasing zinc (Figure 5B,D,E); nonetheless, in the case of Ca^2+^-bound conformers of VILIP1 and GCAP2, we noted a moderate growth in hydrodynamic diameter at high Zn^2+^ concentrations (Figure 5E, bottom panel). However, in the case of NCLD, an increase in Zn^2+^ concentration induced a rapid elevation in particle size for both Ca^2+^-free and Ca^2+^-loaded forms of the protein (Figure 5C). Thus, at 4-fold excess of Zn^2+^, NCLD exists as a dimer, while further increase in zinc content results in the formation of large protein conglomerates (Figure 5C, bottom panel).

**Figure 6:**
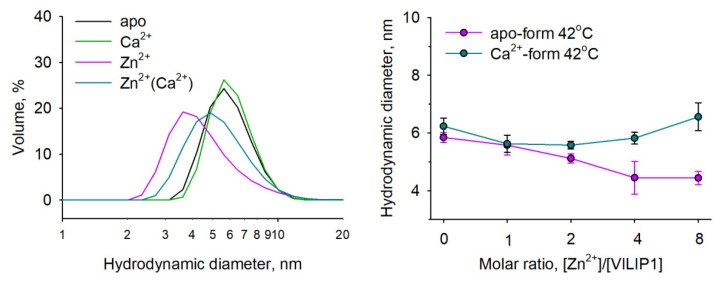
Zn^2+^-dependent dissociation of dimeric VILIP1. Hydrodynamic diameter of VILIP1 at 42 °C was assessed by DLS. Left panel: representative size distribution curves for VILIP1 in the presence of saturating concentration of Ca^2+^, Zn^2+^, both (“Zn^2+^(Ca^2+^)”, or neither (“apo”). Right panel: mean hydrodynamic diameter values for apo- and Ca^2+^-bound VILIP1 in the presence of increasing Zn^2+^ concentrations. Standard error bars were plotted as a result of three independent measurements.

The results of the TS studies suggest Zn^2+^-induced dissociation of VILIP1 dimers at physiological temperatures (see Figure 3B). Therefore, we performed additional DLS studies aimed at verifying this effect. It was found that, at 42 °C, zinc indeed induced a decrease in the hydrodynamic diameter of VILIP1 from 5.8 to 4.4 nm, indicating the transformation of the protein into a monomeric state (Figure 6). The fact that such Zn^2+^-induced changes in the quaternary structure of VILIP1 occur at physiological temperatures could have important functional implications.

Taken together, these findings demonstrate that zinc regulates the dimeric states of recoverin and VILIP1 in an opposite manner, and induces dramatic destabilization and aggregation of NCLD.

### 3.5. Potential Zn^2+^-Binding Sites of NCS Proteins

**Figure 7:**
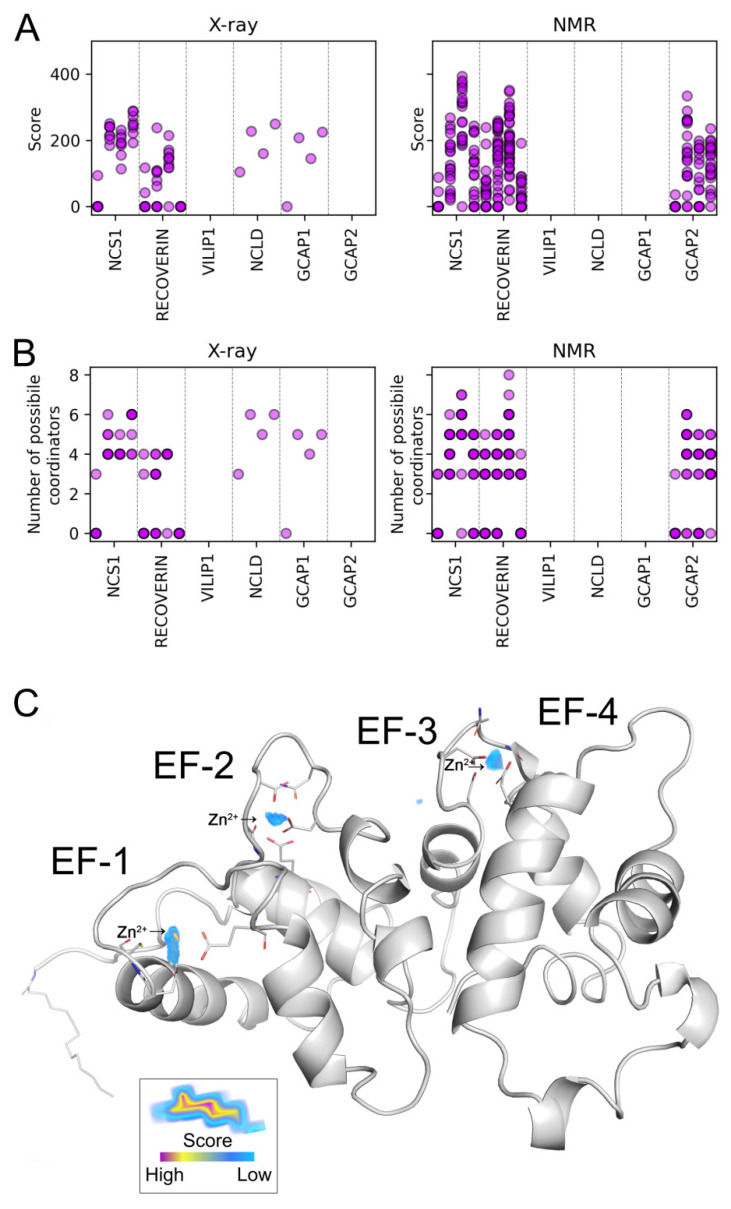
Complementarity of EF-hands to zinc ions estimated based on NMR and X-ray crystal structures of NCS proteins presented in PDB. (**A**) Diagram illustrating the maximum sum score for Zn^2+^ binding in EF-hands 1–4 (left to right) in each NCS protein. The data are presented as semi-transparent dots (magenta) that increase in intensity upon superposition. Zero score indicates “no binding”. The absence of points on the plot indicates the lack of a respective structure in PDB. One coordinator can contribute up to 100 units. (**B**) Number of potential coordinators in a maximum sum score point. Each atom (e.g., OD1/2 in aspartic acid) was counted independently. (**C**) NMR structure of recoverin (PDB ID 1JSA, model 1) with putative Zn^2+^-binding areas in EF1–EF3 (Zn^2+^). The contributing residues are shown as stick-like offshoots. Density of putative positions of Zn^2+^ is visualized as volumetric data, from low (blue) to high (red) values. The highest scoring area is typically located at the middle of the density (the inset).

Our results indicate that each NCS exhibits a unique mode of interaction with zinc, suggesting different amounts and structures of Zn^2+^-binding sites on these proteins. Previously, we developed a structural bioinformatics approach to allow us to identify and rank the putative Zn^2+^-binding areas in proteins based on analysis of the respective sites presented in PDB. Using this approach, we have predicted that in NCS1 Zn^2+^ coordination can occur in the EF-hands, which are characterized as having the highest densities of the metal-chelating groups [36]. Considering these findings, we applied the same approach to compare zinc complementarity with the EF-hands of all NCS proteins, including recoverin, NCLD, GCAP1, and GCAP2, as well as NCS1, which was used as a positive control. Since apo-forms of these proteins are mostly unresolved, the prediction was performed for their Ca^2+^-bound conformers deprived of calcium, which reflect protein states with the maximal density of the metal coordinators. The analysis involved all orthologues resolved by both X-ray and NMR studies. No such structures were found for VILIP1, so it was excluded from consideration. Unexpectedly, we observed a striking difference between the properties of Zn^2+^-binding sites in X-ray and NMR structures (Figure 7A). The difference could be explained by the tight packaging in X-ray structures and artificially high salt concentrations used for crystallization, while NMR structures provide a better reflection of the intrinsic dynamics of EF-hands capable of coordinating a variety of divalent cations in solution [55].

Based on NMR structures, we predicted that NCS1 and recoverin would have similar modes of zinc coordination, with most favorable binding in EF3 (Figure 7B). This finding is supported by our current and previous experimental data [36], which demonstrate similar high-affinity Zn^2+^ binding by both proteins. Moreover, zinc facilitates the dimerization of recoverin, as was previously demonstrated for NCS1 [37]. Notably, both proteins can coordinate zinc ions in all EF-hands (Figure 7A,C). Thus, the single Zn^2+^ detected in Ca^2+^-loaded recoverin (Table 1) can actually be located at EF1 or EF4. In contrast, GCAP2 exhibited an alternate mode of Zn^2+^ binding at three potential sites in EF2–EF4, with the most favorable coordination in EF2. Thus, the results of our structural analysis suggest an attribution of NCS1/recoverin and GCAP2 into different categories concerning the mode of Zn^2+^ binding, and provide further evidence in support of the coordination of this metal in the EF-hands.

## 4. Discussion

The results of this study, together with our previous data [35,36], indicate that most of the NCS proteins are capable of binding Zn^2+^, but display different affinities and stoichiometries for binding, as well as different structural and, potentially, functional outcomes. The sensitivity of NCSs to Zn^2+^ was initially discovered in the case of recoverin, using intrinsic (tryptophan) fluorescence, circular dichroism, and differential scanning calorimetry studies [35]. It was demonstrated that both Ca^2+^-free and Ca^2+^-loaded recoverin bind Zn^2+^ (with K_D_ of 30 μM and 7.1 μM correspondingly), and the latter is moderately destabilized by Zn^2+^ binding, while exhibiting increased affinity to photoreceptor membranes. These data are in good agreement with our current findings, although the affinity of the Zn^2+^ binding to both forms in the current study was refined upwards, which makes it more physiologically relevant (see below). Zn^2+^ associates with both Ca^2+^-free and Ca^2+^-loaded forms of recoverin, and induces noticeable conformational changes in both forms, which manifest as an elevation in the I_350_/I_330_ ratio, hydrodynamic diameter, and temperature of denaturation of the apo-form, and moderate decrease in I_350_/I_330_ and thermal stability of the Ca^2+^-bound form (this study), as well as an increase in α-helical content and surface hydrophobicity of the protein [35]. Thus, one can expect that the binding of zinc ions might affect the signaling activities of both states of recoverin. For instance, Zn^2+^ can promote membrane association of Ca^2+^-free recoverin [35], thereby changing the mode of GRK1 regulation, or induce its dimerization, which may the improve Ca^2+^ sensitivity of the protein, thus altering its function as a Ca^2+^ sensor [56].

A similar mode of high-affinity Zn^2+^ binding was observed in the case of VILIP1 (this study) and NCS1 [36]. These proteins regulate multiple targets and participate in a broad range of signaling pathways (see Section 1) [6,7,8]. NCS1 coordinates Zn^2+^ both as an apo-conformer and a Ca^2+^-loaded conformer, which allows Zn^2+^ to modulate its Ca^2+^-induced signaling activity towards, for instance, GRK1 or D2R. Moreover, same as with recoverin here, Zn^2+^ can promote the formation of a disulfide dimer, which is characterized by a 20-fold increased affinity to GRK1, and an improved inhibitory function towards the enzyme [36,37]. In contrast to recoverin and NCS1, VILIP1 becomes generally insensitive to Zn^2+^ in the presence of Ca^2+^, indicating that, in cells, it could respond to Zn^2+^ only in the absence of calcium signals. Since, according to our nanoDSF study, Ca^2+^-loaded and Zn^2+^-loaded forms of VILIP1 are conformationally distinct, it can be speculated that the binding of these metal ions by the protein should have different functional effects. Notably, it is widely accepted that, under physiological conditions, VILIP1 permanently exists as a functional dimer [53], but we provide the first indications that, in the presence of zinc, it can actually exist in a monomeric form. Indeed, we have observed the dissociation of the dimer directly, in our DLS study, as well as indirectly, from its structural transition at physiological temperature, observed using the TS assay. Thus, our data suggest that, in neurons, VILIP1 can reversibly transfer from a dimeric to a monomeric form (and vice versa) in response to Zn^2+^ binding, which can introduce novel modes of function for this multitarget protein.

**Figure 8:**
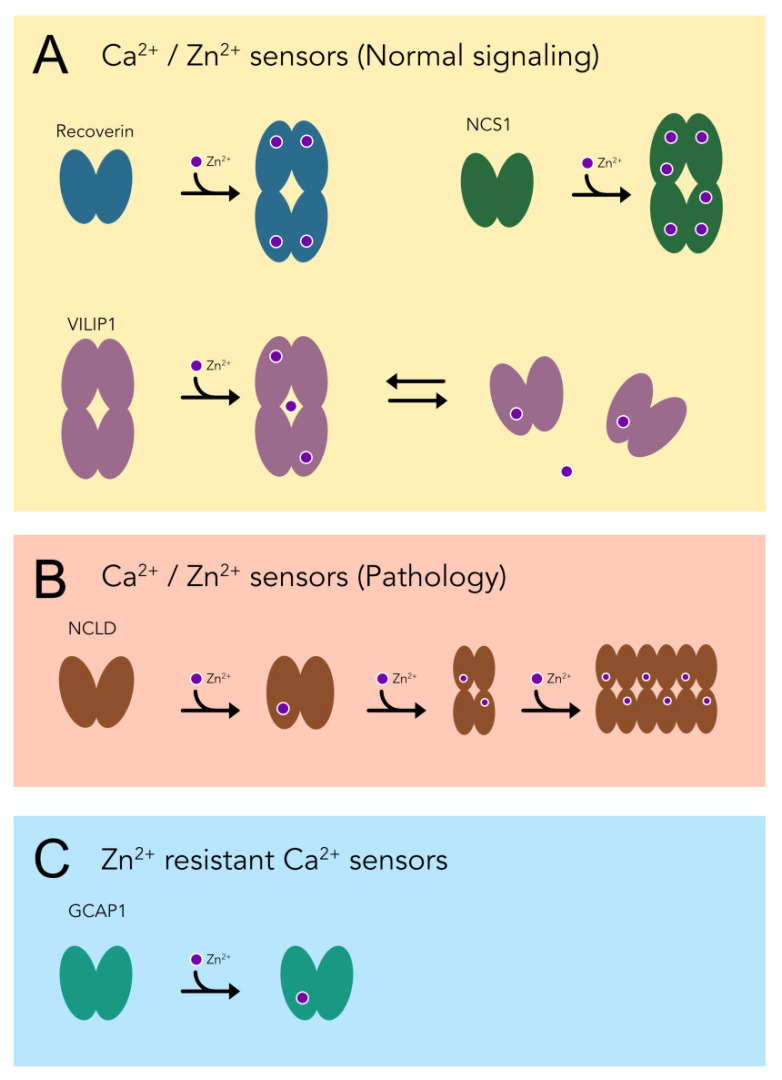
Categorization of NCS proteins based on Zn^2+^-binding properties. (**A**) Physiological Ca^2+^/Zn^2+^ sensor proteins, which bind zinc with high affinity and transform into a new functional state. (**B**) Pathological Ca^2+^/Zn^2+^ sensors responding only to aberrantly high free zinc concentrations by denaturation and aggregation. (**C**) Zn^2+^-resistant, Ca^2+^ sensor proteins. Zinc ions are shown as purple circles.

An open question is whether the revealed Zn^2+^–NCS complexes, characterized by nanomolar and submicromolar binding constants, are relevant to normal cell signaling, or are formed only upon aberrant elevations in Zn^2+^. Indeed, due to high neurotoxicity of free Zn^2+^, its intracellular concentration is tightly regulated, being confined within the subnanomolar range by the concerted action of Zn^2+^-buffer proteins, such as metallothioneins, and homeostatic mechanisms involving Zn^2+^-transporting and Zn^2+^-importing proteins (ZnTs and ZIPs) uploading the metal into mitochondria, lysosomes, and/or vesicles [38]. However, the overall concentration of Zn^2+^ in neural tissue is relatively high (a few hundred micromolar), and a substantial part of this pool may exist as exchangeable (“labile” or “loosely bound”) Zn^2+^, which may possess a signaling function [41,57]. This is especially relevant for the retina, which is highly enriched in Zn^2+^ [41]. The precise quantification of loosely bound Zn^2+^ in neurons is technically challenging, but it was previously suggested to exceed the nanomolar level [57]. In fact, the concentration of free Zn^2+^, released into in the synaptic cleft during neuronal activity reaches 10–300 μM, and this released Zn^2+^ can enter postsynaptic neurons, yielding a local increase in cytosolic concentrations of this metal [38,58,59,60]. Zn^2+^ can permeate cell membranes through glutamatergic receptors, VGCC, and non-selective ionotropic receptors (such as TRP channels), or can be also released from intracellular sources [38,58,61]. Since neurotoxic concentrations of Zn^2+^ were estimated at 0.1–3.0 μM [38], the intracellular sites with comparable or higher affinities can be regarded as a source of exchangeable Zn^2+^ for intracellular signaling. According to our current and previous data [36], such fluctuations in intracellular Zn^2+^ can be detected by NCS1, recoverin, and VILIP1. Thus, these proteins can be classified as physiological Ca^2+^/Zn^2+^ sensors, which may interconnect Zn^2+^ signals with Ca^2+^-regulated pathways (Figure 8A).

In contrast, NCLD seems to be insensitive to Zn^2+^ under normal cellular conditions, as it binds Zn^2+^ with K_D_ > 20 μM. However, if Zn^2+^ concentrations reached this level, it would induce a dramatic destabilizing effect on the structure of NCLD, and promote its prompt aggregation, as is clearly indicated in our nanoDSF and DLS studies. Generally, the highest Zn^2+^ concentration can be generated in the synaptic terminals of glutamate Zn^2+^-releasing (zincergic) neurons, which are enriched in NCLD [62]. So far, there is no direct evidence regarding the pathological conditions associated with NCLD aggregation, as were reported for VILIP1, which forms intracellular aggregates in ALS, and is found in amyloid plaques in patients with AD [12,14]. Interestingly, the aggregation of VILIP1, NCS1, and other NCS proteins is enhanced under oxidative stress conditions, which are commonly characterized by the elevation of free Zn^2+^ released from oxidized cysteine-containing sites in proteins [14,37,63,64,65]. Whether similar effects are realized in the case of NCLD remains an open question, although the silencing of NCLD was demonstrated to have a protective action against spinal muscular atrophy, a neurodegenerative disorder associated with oxidative stress in motor neurons [15,66]. Furthermore, a number of neurological and neuro-ophthalmological disorders, such as cerebral ischemia, AD, ALS, glaucoma, and AMD, involve Zn^2+^-induced cell death [38,39,40,41,42], and NCLD loss due to denaturation and aggregation can contribute to these disorders, as it plays a crucial role in adult neurogenesis [62]. Overall, we can attribute NCLD to pathological Ca^2+^/Zn^2+^ sensors responding only to aberrantly high free Zn^2+^ concentrations by pronounced structural destabilization and aggregation (Figure 8B).

The third group of NCSs, which can be distinguished with respect to Zn^2+^ binding, includes GCAP1 and GCAP2, retina-specific proteins regulating the activity of photoreceptor GCs [5]. GCAPs bind Zn^2+^ regardless of the presence of Ca^2+^, and with moderate conformational changes. According to ITC studies, GCAP1 coordinates single Zn^2+^ with K_D_ of 11–14 μM, while the interaction between Zn^2+^ and GCAP2 does not produce significant calorimetric effects, and can be detected only from nanoDSF studies. Notably, both of these proteins remain structurally stable, and do not aggregate even at millimolar Zn^2+^ concentrations. Thus, although aberrations in the structure of GCAPs are associated with retinal dystrophies and glaucoma [19,20,21], it seems unlikely that the respective pathological effects are linked to their Zn^2+^-dependent properties. Considering these observations, we can categorize GCAP1 and GCAP2 as Zn^2+^-resistant, Ca^2+^ sensor proteins (Figure 8C). Interestingly, a zebrafish GCAP, GCAP5, was previously recognized as a potential Zn^2+^-binding protein, as it contains a common four-cysteine zinc finger motif, and its activity is regulated by divalent transition metal ions [25,67]. In contrast to GCAP5, GCAP1 and GCAP2 lack the cysteine residues forming such a motif, suggesting different Zn^2+^-binding sites, and probably different effects of Zn^2+^ on the activity of these proteins. Zn^2+^ is present in photoreceptor neurons at high levels, and is incorporated within visual cascade proteins, such as rhodopsin and cGMP-phosphodiesterase PDE6 [59,68,69]. Accordingly, it was previously suggested to play a role in the regulation of phototransduction, intracellular signaling in photoreceptors (such as rhodopsin deactivation and regeneration), and/or communication between photoreceptors, other retinal neurons, and/or glial cells [41]. The revealed effects of Zn^2+^ on the structure and function of photoreceptor NCSs, namely recoverin and, to lesser extent, GCAP1 and GCAP2, collectively involved in the regulation of the visual cascade, further supports this idea.

An important issue is the determination of the structural mechanisms underlying the different affinities of NCS proteins to Zn^2+^. The lower Zn^2+^ affinity of recoverin compared to NCS1 could be explained by the presence of a Zn^2+^–myristoyl switch in the former. Indeed, Zn^2+^ produces no effect on membrane binding in NCS1, but increases the membrane affinity of recoverin, suggesting Zn^2+^-induced exposure of the hydrophobic myristoyl to the solution [35,36]. Accordingly, same as for the Ca^2+^–myristoyl switch [22], this would require energy expenditure, which can reduce the affinity of the protein to zinc ions. More significant contributions could be achieved via the structure and the composition of Zn^2+^-binding sites in these proteins. According to our structural predictions, NCS1 and recoverin can coordinate zinc ions in all four EF-hands, including the Ca^2+^-insensitive EF1, with EF3 having the strongest complementarity to Zn^2+^. These findings could explain the similar modes of Zn^2+^ binding and structural responses in these proteins. In turn, GCAP2 displays a notably different pattern of potential Zn^2+^ chelators, with their maximal density in EF2, which further supports the notion of it belonging to a separate category of sensors with regard to Zn^2+^-binding ability.

Taken together, our data demonstrate for the first time that Zn^2+^ binding is a shared property of NCS proteins, which possess different sensitivities to Zn^2+^ and exhibit unique structural outcomes in response to its coordination; this may be of high importance for their normal and pathological signaling in neurons. Changes in the concentration of intracellular free Ca^2+^ regulates a wide variety of neuronal functions, and we suggest that NCS proteins may govern the interconnection between this regulation and Zn^2+^-dependent pathways. Interestingly, death signaling associated with neurological conditions is mediated by both Ca^2+^ and Zn^2+^, with lethal concentrations of approximately 10 μM and 0.1–3.0 μM, respectively. These exact concentrations can be sensed by NCS proteins, suggesting that they may be involved in the pathophysiology of Zn^2+^-mediated neurodegenerative diseases, such as Alzheimer’s disease and glaucoma. Further studies are required for the detection of Zn^2+^–NCS complexes, and the verification of their normal and pathological activities using in vivo models.

## Figures and Tables

**Table 1:** Properties of NCS complexes with zinc ions. Thermodynamic parameters for Zn^2+^/NCS complexes were determined by isothermal titration calorimetry (ITC): the data were fitted using the “one set of sites” model for apo-recoverin, apo-NCLD, Ca^2+^-NCLD, apo-GCAP1, and Ca^2+^-GCAP1, and the “two sets of sites” model for apo-VILIP1. Mid-transition melting temperatures (T_m_) of NCS conformers were determined using thermal shift (TS) assay or nano differential scanning fluorimetry (nanoDSF) approach. Hydrodynamic diameter values were estimated from dynamic light scattering (DLS).

Protein	Ca^2+^	ITC			TS (or nanoDSF)	DLS
		N *	K_D_, μM	ΔH *, kcal/mol	T_m_ *, °C (−/+ Zn^2+^)	D*_h_* *, nm (−/+ Zn^2+^)
recoverin	−	1.8	(1.0 ± 0.2) × 10^−6^	−11.4	50.3/54.8	4.6/5.7
	+	1.3	(2.7 ± 0.3) × 10^−6^	3.0	78.4/69.3	4.6/4.7
VILIP1	−	1.3 0.6	(4.3 ± 1.0) × 10^−8^, (1.7 ± 0.2) × 10^−6^	−2.7 −1.8	68.6/63.7; 38.1	6.3/6.6
	+	no binding			n.d./38.2	6.7/6.9
NCLD	−	0.9	(2.6 ± 0.5) × 10^−5^	−2.4	61.1/38.8	5.4/5.8
	+	2.6	(2.0 ± 0.5) × 10^−5^	−1.4	84.5/51.2	5.8/6.6
GCAP1	−	0.7	(1.4 ± 0.2) × 10^−5^	−9.3	n.d.	5.9/6.8
	+	1.1	(1.1 ± 0.1) × 10^−5^	−6.1	n.d.	5.9/7.3
GCAP2	−	n.d.	n.d.	n.d.	n.d.	6.1/6.4
	+	n.d.	n.d.	n.d.	n.d.	6.1/6.4

* Error did not exceed 10%. n.d.—not determined.
